# Asymmetric Recruitment of β-Arrestin1/2 by the Angiotensin II Type I and Prostaglandin F2α Receptor Dimer

**DOI:** 10.3389/fendo.2019.00162

**Published:** 2019-03-18

**Authors:** Dany Fillion, Dominic Devost, Rory Sleno, Asuka Inoue, Terence E. Hébert

**Affiliations:** ^1^Department of Pharmacology and Therapeutics, McGill University, Montréal, QC, Canada; ^2^Graduate School of Pharmaceutical Sciences, Tohoku University, Sendai, Japan; ^3^Japan Science and Technology Agency, Precursory Research for Embryonic Science and Technology, Kawaguchi, Japan

**Keywords:** G protein-coupled receptor (GPCR), dimerization, allosteric regulation, arrestin, angiotensin, prostaglandin

## Abstract

Initially identified as monomers, G protein-coupled receptors (GPCRs) can also form functional homo- and heterodimers that act as distinct signaling hubs for cellular signal integration. We previously found that the angiotensin II (Ang II) type 1 receptor (AT1R) and the prostaglandin F2α (PGF2α) receptor (FP), both important in the control of smooth muscle contractility, form such a functional heterodimeric complex in HEK 293 and vascular smooth muscle cells. Here, we hypothesize that both Ang II- and PGF2α-induced activation of the AT1R/FP dimer, or the parent receptors alone, differentially regulate signaling by distinct patterns of β-arrestin recruitment. Using BRET-based biosensors, we assessed the recruitment kinetics of β-arrestin1/2 to the AT1R/FP dimer, or the parent receptors alone, when stimulated by either Ang II or PGF2α. Using cell lines with CRISPR/Cas9-mediated gene deletion, we also examined the role of G proteins in such recruitment. We observed that Ang II induced a rapid, robust, and sustained recruitment of β-arrestin1/2 to AT1R and, to a lesser extent, the heterodimer, as expected, since AT1R is a strong recruiter of both β-arrestin subtypes. However, PGF2α did not induce such recruitment to FP alone, although it did when the AT1R is present as a heterodimer. β-arrestins were likely recruited to the AT1R partner of the dimer. Gα_q_, Gα_11_, Gα_12_, and Gα_13_ were all involved to some extent in PGF2α-induced β-arrestin1/2 recruitment to the dimer as their combined absence abrogated the response, and their separate re-expression was sufficient to partially restore it. Taken together, our data sheds light on a new mechanism whereby PGF2α specifically recruits and signals through β-arrestin but only in the context of the AT1R/FP dimer, suggesting that this may be a new allosteric signaling entity.

## Introduction

G protein-coupled receptors (GPCRs) have historically been studied and understood as functional monomers. Although well-established for class-C GPCRs (1, 2), an increasing body of evidence now supports that class-A GPCRs also form dimers (3, 4) and even higher-order oligomers; however, the exact physiological significance, and pharmacological importance of such complexes still remain elusive. Given that GPCRs are one of the most successfully exploited drug targets, the continuing emergence of dimers represent a promising opportunity not only to better understand GPCR biology and their roles in disease etiology, but also to reveal their full therapeutic potential as unique functional entities (5).

One untapped feature of GPCR dimers might be a potential ability to assemble according to cellular needs and to combine two separate, independent orthosteric binding sites—especially true for heterodimers—where one receptor protomer can potentially allosterically modulate the function of the other, and *vice-versa*. Further, GPCR dimers can also allow different intracellular signaling partners to selectively interact with only one of the two protomers or both, expanding the possibilities for the cell to adapt to different conditions. For example, a D2 dopamine receptor homodimer was found to be organized asymmetrically with respect to its G protein partners such that occupation of the first protomer facilitated downstream cellular signaling through the second protomer, while occupation of the latter (or even its constitutive activity) modulated signaling allosterically without inducing a signal on its own (6). In the context of such a functional dimeric assembly, benefits usually attributed to allosteric modulators can be derived from an otherwise orthosteric site, offering novel avenues to fine-tune signaling efficacy, improve receptor subtype selectivity and tissue specificity as well as off target effects (7).

For most class-A GPCR dimers, the minimal signaling unit is thought to be composed of two receptors and one heterotrimeric G protein to form a pentameric structure (3, 4, 6). Interestingly, monomeric GPCRs have recently been found to also form what is called a super-complex, or “megaplex,” by simultaneously binding through its core region with G protein and through its phosphorylated C-terminal tail with β-arrestin (8); therefore, opening the possibility for GPCR dimers to form higher-order oligomeric complexes with both G protein and β-arrestin. Indeed, β-arrestin has been demonstrated to adopt two distinct conformations when bound to an engineered β_2_-adrenergic receptor (β_2_AR) (9). β-arrestin adopts a more engaged “core” conformation in which it contacts extensively with the transmembrane domains, the intracellular loops along with the phosphorylated C-terminal tail of the receptor, and a more relaxed conformation (“core” conformation) in which it contacts with the receptor C-terminal tail only, allowing β-arrestin to more freely hang from the receptor and exposing the entire intracellular surface (9). Some crystal structures of GPCRs also appear to support the formation of “megaplex.” An arrangement similar to the “core” conformation have been reported in a rhodopsin/visual arrestin crystal structure (10), while the β_2_AR/Gs protein crystal structure seems compatible with the “tail” conformation and the simultaneous presence of G protein and β-arrestin (11).

A hurdle in investigating class-A GPCR dimerization has been the limited capacity to identify the respective function of the protomers within a dimer, that is which protomer binds the ligand and which protomer signals. This minimal functional unit is used to investigate the interplay between β-arrestin 1/2 and multiple G proteins at the larger intracellular interface of a GPCR dimer using a panel of BRET-based biosensors. We previously showed that AT1R and FP could be co-purified together using immunoprecipitation in HEK 293 cells and in vascular smooth muscle cells combined with photoaffinity labeling (12). We noted that FP antagonists resulted in inhibition of Ang II-mediated contraction, an effect that cannot be mediated by second messenger-activated crosstalk via protein kinases, since these ligands did not activate such signaling events. Similar results were obtained when we pre-treated cells with an AT1R antagonist when measuring FP-mediated contraction (12).

We also engineered FlAsH tags and *Renilla* luciferase into both AT1R and FP and co-expressed them with their untagged counterparts (13). We again noted asymmetric transmission of conformational information between protomers. AT1R-induced conformational rearrangements in FP were dependent on both the presence of activatable Gα_q_ as well as the possible involvement of phospholipase Cβ, a proximal Gα_q_-effector. Association of GPCRs, G proteins and effectors likely represent core units of signaling complex organization, which is reflected by mutual allosteric effects. We also showed that AT1R-driven conformational changes in FP were independent of the activation of PKC, a shared downstream receptor signaling pathway. Allosteric interactions occur in the plasma membrane, mediated through a shared G protein in a heterodimer. The AT1R/FP heterodimer remained intact even in the absence of Gα_q_, thus they are not required for assembly of receptor heterodimers rather allosterically connecting the two receptors as part of signaling complexes. With respect to allosteric interactions in the AT1R/FP dimer, AT1R was able to modulate FP conformation but the converse was not true (at least with the biosensors we were using) (14). Further, the AT1R to FP conformational crosstalk in the heterodimer was biased toward Gα_q/11_ as a preferred heterodimer partner, as no conformational effects were observed when Gα_12/13_ or Gα_i_ function or levels were altered. Such a perceived coupling preference in the heterodimer might again be because our initial panels of conformational biosensors may have only been sensitive to the presence of particular G proteins. Here, we extend this finding to β-arrestin to show how this scaffolding/adaptor protein can be symmetrically (e.g., cis-activation) and asymmetrically (e.g., trans-activation) regulated in response to Ang II and PGF2α, respectively.

## Materials and Methods

### Materials

All cell culture media, reagents, and antibiotics were from Wisent Inc. (St-Bruno, Québec, Canada). All DNA primers for molecular cloning were custom-made by Integrated DNA Technologies Inc. (Coralville, IA, USA). All enzymes and other materials used for molecular cloning were from New England Biolabs Ltd. (Ipswich, MA, USA), except for the Pvu II and Taq I restriction enzymes that were both from Takara Bio Inc. (Noji Higashi, Kusatsu Shiga, Japan). Cell transfection reagent was from Invitrogen, Thermo Fisher Scientific Inc. (Waltham, MA, USA). All chemicals, including Ang II and the AT1R antagonists were from Sigma-Aldrich Inc. (St. Louis, MO, USA) unless otherwise specified. PGF2α and cloprostenol were from Cayman Chemical Company (Ann Arbor, MI, USA). Coelenterazine h was from NanoLight Technologies (Pinetop, AZ, USA).

### CRISPR-Gene Deletion Lines

As recently reported (14), the HEK 293 ΔGα_q/11/12/13_ cell line wherein all the genes encoding for G_q_, G_11_, G_12_, and G_13_ proteins have been knocked out was generated by simultaneously targeting the GNA12 and the GNA13 genes of the previously established HEK 293 ΔGα_q/11_ cells (15), using CRISPR/Cas9 as described previously (16).

### Molecular Cloning and Mutagenesis

The following plasmids pcDNA3/SP-FLAG-hAT1R-WT (12), pcDNA3.1/SP-FLAG-hAT1R-RlucII (17), pcDNA3/SP-HA-hFP-RlucII (18), β-arrestin 1-RlucII (19, 20), β-arrestin 2-RlucII (19, 20) were used. A K44 dynamin mutant in pcDNA3 was a generous gift from Dr. Denis Dupré (Dalhousie University). The different pcDNA3.1 plasmids encoding Gα_q_, Gα_11_, Gα_12_, Gα_13_ were from UMR cDNA resource center (www.cdna.org; University of Missouri-Rolla, Rolla, MO, USA). The pcDNA3.1/SP-FLAG-hAT1R-KPVAT-Venus plasmid was created from pcDNA3/SP-FLAG-hAT1R-WT that was amplified by PCR using the forward primer 5′-CCTAGCTAGCTCGAGGCCACCATGAACACGATCATC-3′ and reverse primer 5′-TACCGGTGGCGACCGGTTTCTCAACCTCAAAACATGGTGC-3′ (without a stop codon). The purified PCR fragment obtained was then subcloned in-frame into the NheI and AgeI restriction sites located in 5′ and 3′, respectively, of pcDNA3.1/Venus, which was a kind gift from Dr. Michel Bouvier (Université de Montreál, Montréal, Canada). In a same way, the pcDNA3.1/SP-FLAG-hAT1R[Δ325]-KPVAT-Venus plasmid was generated using the forward primer 5′-CCTAGCTAGCTCGAGGCCACCATGAACACGATCATC-3′ and reverse primer 5′-AAAGGGTGGCGACCGGTTTGGCTTTTGGGGGAATATATTTTAGAAGCTG-3′ (without a stop codon) to amplified pcDNA3/SP-FLAG-hAT1R-WT, and the resulting PCR fragment was then subcloned into the same restriction sites of pcDNA3.1/Venus. The HA-hOTR-Venus plasmid was made by the PCR amplification of a previously described hOTR-YFP construct (21) using the forward primer 5′-TTATGCCTGCGGATCCGAGGGCGCGCTCGCAGCCAACT-3′ and reverse primer 5′-CGCCACCTCCGGATCCCGCCGTGGATGGCTGGGA-3′. The purified PCR fragment obtained was then digested at the BamHI restriction site, and subcloned in-frame by recombination into pIRESpuro3/HA-Venus using the In-Fusion cloning system from Clontech Laboratories (Mountain View, CA, USA). The pcDNA3.1/SP-HA-hFP-WT plasmid was prepared by two rounds of PCR. First, a previously described pIRESpuro3/HA-hFP-WT construct (18) was amplified using the forward primer 5′-ATGAACACGATCATCGCCCTGAGCTACATCTTCTGCC-3′ and reverse primer 5′-ATCCGAATTCCTAGGTGCTTGCTGATTTCTCTGC-3′. The purified PCR fragment was submitted to a second round of PCR amplification using the forward primer 5′-CCTAGCTAGCTCGAGGCCACCATGAACACGATCATC-3′ and the same reverse primer. The resulting PCR fragment was then purified and digested at the XhoI and EcoRI restriction sites located at the 5′ and 3′, respectively, and subcloned into pcDNA3.1 from Invitrogen (Waltham, MA, USA). The pcDNA3.1/SP-HA-hFP-KPVAT-Venus plasmid was produced using the forward primer 5′-CCTAGCTAGCTCGAGGCCACCATGAACACGATCATC-3′ and reverse primer 5′-GGTGGCGACCGGTTTGGTGCTTGCTGATTTTGC-3′ to amplify pcDNA3.1/SP-HA-hFP-WT by PCR. The purified PCR fragment was then digested at the NheI and AgeI restriction sites located in 5′ and 3′, respectively, and subcloned into pcDNA3.1/Venus. All constructs were verified by bidirectional DNA sequencing. The constructs for enhanced bystander BRET to examine AT1R and FP trafficking, Lyn-rGFP, and rGFP-FYVE were used as previously described (17).

### Cell Culture and Transient Transfection

The HEK 293 parental and ΔGα_q/11/12/13_ cell lines were cultured in 75 cm^2^ plastic flasks containing Dulbecco's Modified Eagle's Medium (DMEM) high glucose supplemented with 5% (v/v) fetal bovine serum (FBS) and 1% (w/v) penicillin–streptomycin (P-S) antibiotics. Cells were cultured in a controlled, humidified environment maintained at 37°C and 5% CO_2_ atmosphere until a confluency of 80–100% was reached. For transfection, cells were trypsinized, and 125,000 cells/well in 1 mL of the same fresh media were seeded into 12-well plates. Cells were incubated overnight in at 37°C. After 24 h, media from the plate was replaced by 1 mL/well of DMEM supplemented with 5% (v/v) FBS. Cells were transfected with the appropriate plasmids encoding for AT1R (0.3 μg/well), FP (0.1 μg/well), and the sensors (β-arrestin 1/2-RlucII (0.0025–0.025 μg/well), along with pcDNA3.1(-) for a total of 1.0 μg per well using Lipofectamine® 2000 [2.5(Lipo2000):1(DNA) ratio] and following the manufacturer's instructions. Twenty-four hours post-transfection, the cells were trypsinized, and 40,000–50,000 cells/well were transferred into white 96-well plates (Costar® #3917, Corning, NY, USA) previously coated with poly-L-ornithine hydrobromide (Sigma-Aldrich Inc., St. Louis, MO, USA). Cells were left in a cell culture incubator for another 24 h before performing the experiments. Mycoplasma testing was carried out periodically on all cell lines using the MycoAlertTM kit from Lonza (Rockland, MD, USA).

### Bioluminescence Resonance Energy Transfer (BRET) Assay

BRET experiments were performed as described elsewhere (13, 14, 18, 22, 23). For intermolecular BRET experiments, energy-donor proteins were fused to *Renilla* luciferase (Rluc) II and energy-acceptor proteins were fused to the yellow florescent protein (YFP). In all experiments, coelenterazine h was used as the Rluc/RlucII substrate to generate light with a maximal emission peak at 480 nm, allowing YFP excitation. In a typical experiment, transfected cells from a white 96-well plate were carefully washed once with 150 μL of Kreb's buffer [146 mM NaCl, 4.2 mM KCl, 0.5 mM MgCl_2_, 1 mM CaCl_2_, 5.9 mM glucose, and 10 mM HEPES pH 7.4, 0.1% (v/v) glucose], and then the cells were incubated in the absence (i.e., with only Kreb's buffer alone) or presence (i.e., with AT_1_R antagonists or endocytosis inhibitors) of pretreatments in 95 μL/well of the same fresh Kreb's buffer. The plate was protected from light, and the cells were incubated for 1 h at 37°C for BRET experiments which were conducted at 37°C. A TriStar^2^ LB 942 multimode microplate reader from Berthold Technologies Inc. (Bad Wildbad, 75323, Germany) equipped with the predetermined BRET1 filter pair F485/F530, and a VICTOR X-light multilabel plate reader from Perkin Elmer Inc. (Waltham, MA, USA) equipped with the predetermined BRET1 filter pair F460/F535 nm (200 msec./filter, alternatively) were used to measure BRET ratios. For enhanced bystander BRET we used 410 nm (donor) and 515/40 nm (acceptor) filters. Under these conditions, both plate readers were able to complete one cycle of BRET ratio measurement in 2 min for a 96-well plate. Experiments with 96-well plates were also carried out at once following four consecutive steps in a timely fashion so that a same time interval apply between each well throughout the process: (1) adding the coelenterazine h substrate for light generation (using a repeater pipette); (2) measuring the BRET ratios of basal, unstimulated cells; (3) stimulating the cells with either vehicle or ligand (using a multichannel pipette); (4) measuring the BRET ratios of stimulated, ligand-induced cells. Following a 1 h incubation in Krebs buffer, 25 μL/well of a 30 μM coelenterazine h solution (for a final concentration of 5 μM) was sequentially added to the cells so it takes 2 min to fill up the entire 96-well plate. The basal, unstimulated BRET ratios were then immediately measured over a period of 10 min (5 measuring cycles). Upon completion, the 96-well plate was rapidly removed from the plate reader, and 30 μL/well of either vehicle or 5 μM ligand (e.g., Ang II or PGF2α, for a final concentration of 1 μM in 150 μL) was sequentially added to the cells so, again, it takes 2 min to fill up the entire 96-well plate. The ligand-induced BRET ratios were then immediately measured over a period of 90 min (45 measuring cycles).

### Data Analysis

**Figure 1:**
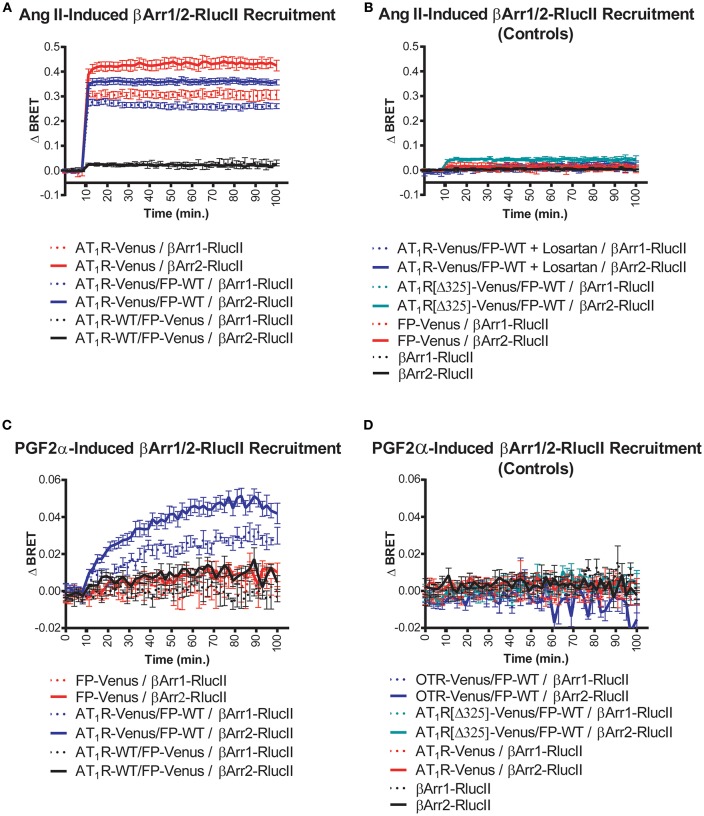
Symmetric and asymmetric recruitment of β-arrestin 1/2 to the AT1R/FP dimer by Ang II and PGF2α, respectively. **(A)** Ang II (1 μM) induced a rapid and sustained symmetric recruitment of β-arrestin 1-RlucII (dashed lines; t_1/2_ < 2 min.) and β-arrestin 2-RlucII (full lines; t_1/2_ < 2 min) to AT1R-Venus alone (purple lines) and to a lesser extent, the AT1R-Venus/FP-WT dimer (blue lines), but not to the AT1R-WT/FP-Venus dimer (black lines). **(B)** No and very small responses were detected with Ang II by blocking AT1R-Venus with losartan (100 μM) (dashed and full blue lines), and when an AT1R[Δ325]-Venus mutant deficient for both β-arrestin 1/2-RlucII recruitments (dashed and full blue-green lines) were used in the context of the dimer, respectively. Responses were all specific to AT1R since no responses were detected for FP-Venus alone (dashed and full red lines) and β-arrestin 1/2-RlucII alone (dashed and full black lines). **(C)** PGF2α (1 μM) induced a slower, yet sustained asymmetric recruitment of βarr1-RlucII (dashed blue lines; t_1/2_ = 20 min) and β-arrestin 2-RlucII (full blue lines; t_1/2_ = 17 min) to the AT1R-Venus/FP-WT dimer only, but not to FP-Venus alone (dashed and full red lines) and the AT1R-WT/FP-Venus dimer (dashed and full black lines). **(D)** No responses were detected with PGF2α when OTR-Venus (dashed and full blue lines) and an AT1R[Δ325]-Venus mutant deficient for both β-arrestin 1/2-RlucII recruitment (dashed and full blue-green lines) were used in the context of the dimer. Responses were all specific to FP since no responses were detected for AT1R-Venus (dashed and full red lines) and β-arrestin 1/2-RlucII alone (dashed and full black lines). Data represent mean ± s.e.m. of independent experiments [biological replicates, each performed in triplicate (*n* = 3–15)].

**Figure 2:**
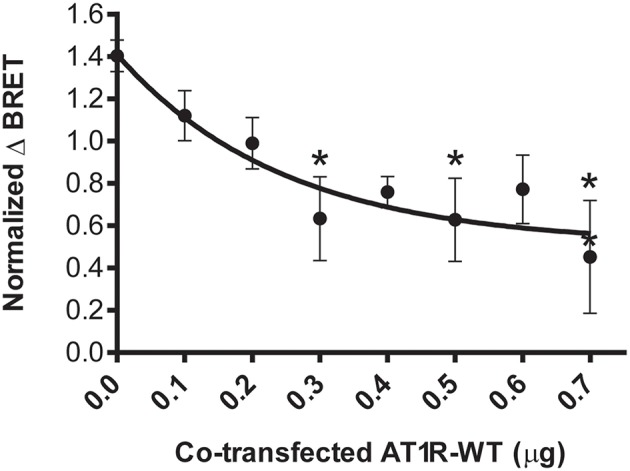
Specificity of the PGF2α-induced β-arrestin 2 recruitment to the AT1R/FP dimer. The recruitment of β-arrestin 2-RlucII to the AT1R-Venus (0.3 μg)/FP-WT (0.1 μg) dimer (fixed amount) induced by PGF2α (1 μM) was titrated by co-transfecting increasing amounts of AT1R-WT (0.0–0.7 μg). Increased expression of AT1R-WT competed with AT1R-Venus for dimerization with FP-WT, reducing the capacity of the AT1R-Venus/FP-WT dimer to recruit β-arrestin 2-RlucII. Data were normalized for AT1R-Venus expression and represent mean ± s.e.m. of independent experiments [biological replicates each performed in triplicate (*n* = 3–5)]. ^*^p < 0.05, compared to AT1R-WT (0.0 μg).

BRET ratios were calculated as BRET = λ_530or535_/λ_485or460_, and ligand-induced BRET changes as ΔBRET = BRET_Ligand_ − BRET_Vehicle_. Conditions associated with ligand and vehicle were performed in triplicate (i.e., 3-wells each) for each biological replicate, and averaged data were used in all calculations. As shown in the figures, BRET data were reported as kinetic traces or bar graphs integrating the area under the curve. Statistical analysis and curve fitting were all carried out using the GraphPad PRISM software v6.0 (La Jolla, CA, USA). For Figure 1, half-time values were calculated using a one-phase association equation and a least squares method to fit the data on a curve by non-linear regression. For Figure 2, ΔBRET data were fit on a curve by non-linear regression using a one-phase exponential dissociation decay and a least squares method. Statistical analysis on normalized ΔBRET data was performed using a Mann-Whitney *U*-test (*p* < 0.05), followed by a *post-hoc* Dunn's test to correct for multiple comparisons (all conditions vs. 0 μg). For **Figure 4** statistical analysis on ΔBRET data was performed using an unpaired Student's *t*-test (*p* < 0.01), followed by a *post-hoc* Bonferroni test to correct for multiple comparisons (all conditions vs. PGF2α or cloprostenol). EC_50_ values from Table 1 were calculated with the following three parameter equation: Response = Bottom + [(Top - Bottom) / (1 + 10(logEC_50_ − log[A])], where Top and Bottom represented maximal and minimal asymptotes of the curve, [A] is the agonist concentration expressed in (M) and EC_50_ is the agonist concentration (M) that generated a response half way between the top and bottom.

**Table 1:** EC_50_ values for β-arrestin 2-RlucII recruitment to the AT1R-Venus/FP-WT dimer.

	**EC_**50**_**
	**Mean ± SEM**
PGF2α	7.2 × 10^−9^ ± 6.9 × 10^−10^
AL-8810	3.0 × 10^−6^ ± 1.6 × 10^−6^
Cloprostenol	1.8 × 10^−10^ ± 5.2 × 10^−11^
Fluprostenol	8.9 × 10^−10^ ± 7.2 × 10^−11^
Latanoprost	6.1 × 10^−9^ ± 1.2 × 10^−9^
Tafluprost	4.8 × 10^−10^ ± 1.1 × 10^−10^

*HEK 293 parental cells transiently expressing β-arrestin 2-RlucII, AT1R-Venus, and FP-WT dimer were assayed as described in section Materials and Methods. Data represent mean ± s.e.m. of independent experiments performed in triplicate (n = 3)*.

## Results

### Symmetric and Asymmetric β-Arrestin Recruitment Mediated by Ang II and PGF2α, Respectively

We used BRET-based biosensors to examine real-time recruitment of β-arrestin 1/2-RlucII to AT1R-Venus alone, FP-Venus alone, and the AT_1_R/FP dimer wherein Venus was fused to either AT1R or FP. Ang II stimulation induced a rapid, robust, and sustained β-arrestin 1/2-Rluc recruitment to AT_1_R-Venus alone (Figure 1A) typical of a class-B GPCR interaction with β-arrestin (24). While showing an identical kinetic profile, co-expression of FP-WT to form the putative AT1R-Venus/FP-WT dimer dampened the extent of β-arrestin recruitment (Figure 1A). However, we cannot say with certainty that this effect is not due to reduced AT1R expression in the presence of co-expressed FP or simply a different physical spacing of BRET donor and acceptor caused by formation of the heterodimer. β-arrestin recruitment could blocked by the AT1R antagonist losartan and was not detected using a mutated AT1R[Δ325]-Venus with a markedly reduced ability to recruit β-arrestin (Figure 1B). On the other hand, in the AT1R-WT/FP-Venus dimer configuration, however, Ang II failed to recruit β-arrestin 1/2-Rluc (Figure 1A). Altogether, these findings suggest that Ang II occupancy of the AT1R/FP dimer induced a recruitment of β-arrestin 1/2 that is symmetric, or *cis*, to the AT1R protomer, and that FP may act to reduce this response. By way of comparison, PGF2α stimulation did not induce recruitment of β-arrestin 1/2-Rluc to FP alone, as previously shown (25, 26), or to the AT1R-WT/FP-Venus dimer (Figure 1C). Interestingly, however, it induced a comparatively slower, less robust, yet prolonged β-arrestin 1/2-Rluc recruitment to the AT1R-Venus/FP-WT than Ang II did, suggesting this time that the PGF2α occupancy of FP recruits β-arrestin 1/2 to its AT1R partner protomer in an asymmetrical, or *trans*, fashion (Figure 1C). Recruitment of β-arrestin was not seen when FP-WT was co-expressed in the context of a dimer with β-arrestin 1/2 recruitment-deficient AT1R[Δ325]-Venus mutant or another peptidergic class-A GPCR, the oxytocin (OT) receptor (OTR, Figure 1D). We previously showed that OTR does not dimerize with FP (13) so results presented here are consistent with this earlier study. In all cases, β-arrestin 2 was shown to be a better responder than β-arrestin 1 for both Ang II and PGF2α stimulation (Figures 1A,C). All responses were also specific since no recruitment was detected when AT_1_R alone or FP alone were stimulated with their non-cognate ligands (Figures 1B,D), or when β-arrestin 1/2-RlucII were transfected alone. Furthermore, the PGF2α-induced recruitment of β-arrestin 2-RlucII to the AT1R-Venus/FP-WT dimer was titrated by coexpression of increasing amounts of AT1R-WT to disrupt the reporting dimer by competition, demonstrating the specificity of AT1R-Venus/FP-WT dimer formation critical for asymmetric recruitment of β-arrestin 2 (Figure 2).

**Figure 3:**
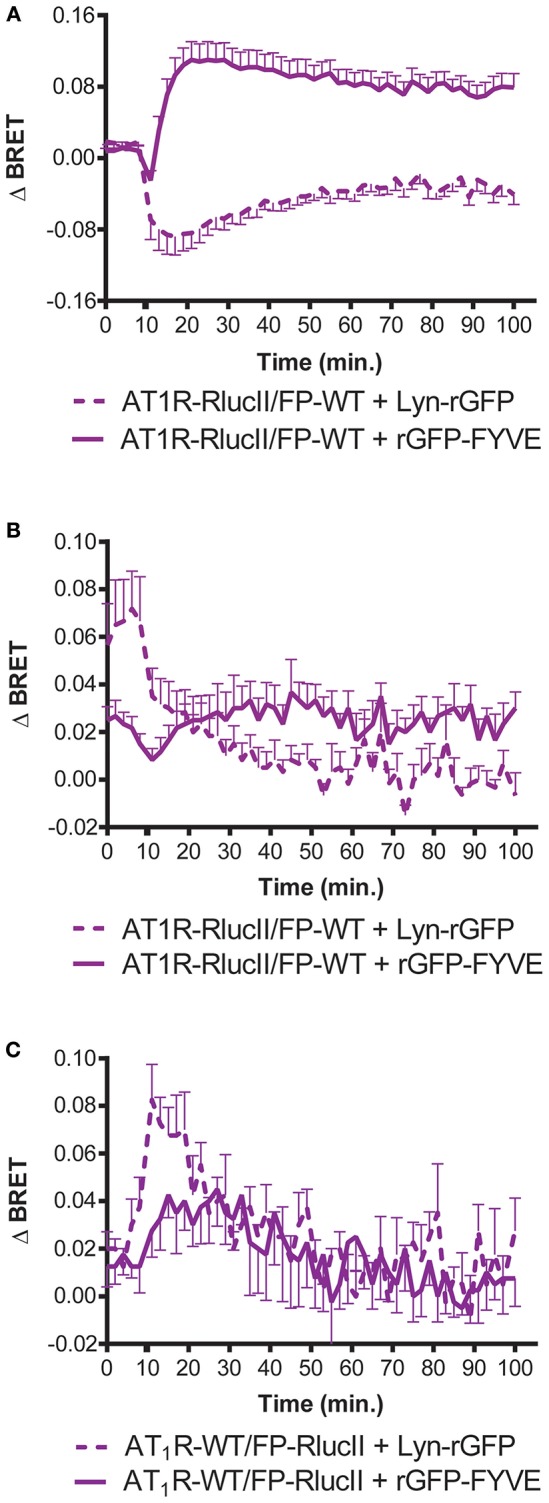
Enhanced bystander BRET shows FP can stimulate AT1R internalization. HEK293 cells were transfected with the indicated enhanced BRET pairs. To monitor AT1R- or FP-mediated trafficking of AT1R-RlucII from the membrane (loss of Lyn-rGFP bystander BRET) or endosomal trafficking (as measured by increase in rGFP-FYVE bystander BRET), AT1R-RlucII were co-transfected with wildtype FP and the indicated bystander BRET partner. Cells were stimulated with **(A)** 1 μM Ang II or **(B)** 1 μM PGF2α and monitored over time. **(C)** FP-RlucII were co-transfected with wildtype AT1R and the indicated bystander BRET partner and stimulated with 1 μM PGF2α and monitored over time. BRET signals were measured as described in Materials and Methods. Data represent mean ± s.e.m. of independent experiments performed in triplicate (*n* = 3).

In order to provide an independent measure of how the dimer resulted in a new ability for FP to induce trafficking of the AT1R/FP dimer, we used enhanced bystander BRET (17) to follow association of AT1R-RlucII or FP-RlucII with a membrane BRET acceptor (Lyn-rGFP) or early endosome acceptor (rGFP-FYVE). In the case where AT1R-RlucII was co-expressed with WT FP and either of the BRET acceptors, stimulation with either Ang II (Figure 3A) or PGF2α (Figure 3B) led to a time-dependent loss of the membrane signal and an increase in the endosomal population. When FP-RlucII was co-expressed with WT-AT1R and the two BRET acceptors, again PGF2α induced a loss of membrane-localized receptor and a transient increase in an early endosomal population (Figure 3C). This suggests that the two ligands might drive receptors into different trafficking itineraries, discussed below. We also attempted to measure what would happen when we stimulated this combination of receptors (FP-RlucII and WT-AT1R) with Ang II. There was little change in bystander BRET for either location-specific biosensor likely because in our hands the AT1R expresses so much better than the FP and thus most of the AT1R in this configuration was not coupled to FP (data not shown).

Next, we assessed to what extent the AT1R-Venus/FP-WT dimer could asymmetrically recruit the β-arrestin 2-Rluc by examining a panel of five chemically distinct FP ligands, some of which have clinical indications (27). Except for AL-8810, all the ligands we tested, cloprostenol, fluprostenol, latanoprost, and tafluprost, were found to recruit β-arrestin 2-Rluc in an asymmetric fashion with EC_50_ values in the low nanomolar to the picomolar range (Table 1). While all the other tested ligands exhibit full agonist properties, AL-8810 is the only one shown to display partial agonist properties (28), and had an EC_50_ value for β-arrestin 2-RlucII recruitment in the micromolar range (Table 1). Further, as AL-8810 promotes activation of ERK1/2 by transactivation of the epidermal growth factor receptor (25), this may be possible explanation for this discrepancy. It is clear, however, that a number of FP ligands can recruit β-arrestin to the dimer.

**Figure 4:**
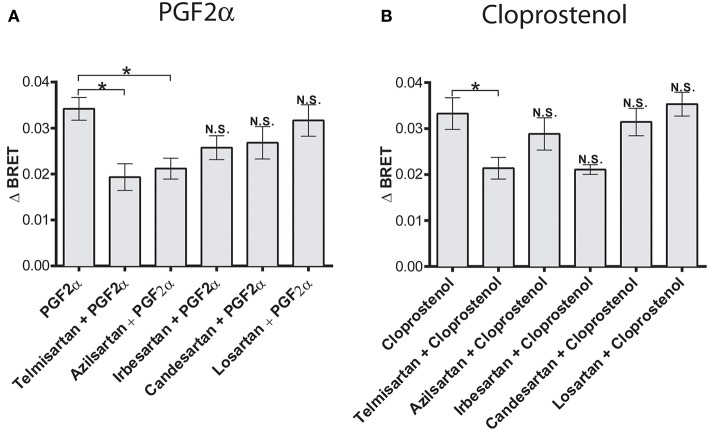
Biased allosteric regulation of the PGF2α- and cloprostenol-induced β-arrestin2 recruitment to the AT_1_R/FP dimer by AT1R antagonists. **(A)** Pretreatment (1 μM for 30 min) with telmisartan or azilsartan allosterically inhibited recruitment of β-arrestin 2-RlucII to the AT1R-Venus/FP-WT dimer in response to PGF2α (1 μM). Under the same experimental conditions, irbesartan, candersartan and losartan produced no allosteric effects. **(B)** Pretreatment (1 μM for 30 min) with telmisartan allosterically inhibited the recruitment of β-arrestin 2-RlucII to the AT1R-Venus/FP-WT dimer in response to cloprostenol (1 μM). Under the same experimental conditions, azilsartan, irbesartan, candersartan and losartan produced no allosteric effects. Data represent mean ± s.e.m. of independent experiments [biological replicates performed in triplicate (*n* = 3–6)]. ^*^p < 0.05. N.S., non-significant.

Having previously shown allosteric crosstalk between FP and AT1R in that antagonists for either receptor modulated signaling via the partner receptor, we next tested how different AT1R antagonists modulated PGF2α-mediated recruitment of β-arrestin to the dimer. PGF2α responses were regulated by both temilsartan and azilsartan (Figure 4A), while cloprostenol responses were regulated by temilsartan only (Figure 4B). On the other hand, other AT1R antagonists we tested, irbesartan, candersartan, and losartan, did not allosterically modulate the responses induced by either PGF2α or cloprostenol.

### G Proteins Alter the Extent of β-Arrestin Recruitment

**Figure 5:**
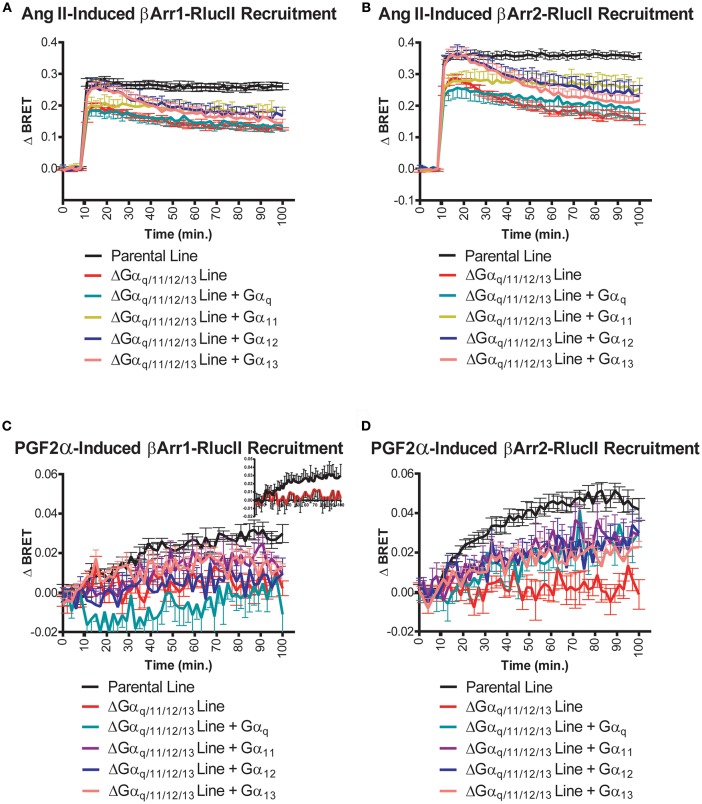
Role of G proteins in Ang II- and PGF2α-induced β-arrestin 1/2 recruitment to the AT1R/FP dimer. **(A,B)** Compared to the HEK 293 parental cell line (black lines), Ang II (1 μM) induced a decaying and much lower, yet sustained recruitment of β-arrestin 1/2-RlucII to the AT1R-Venus/FP-WT dimer when evaluated in the HEK 293 ΔGα_q/11/12/13_ cell line (red lines). **(C,D)** PGF2α (1 μM) induced the recruitments of β-arrestin 1/2-RlucII to the AT1R-Venus/FP-WT dimer in the HEK 293 parental cell line (black lines) only, and virtually no responses were detected with the HEK 293 ΔGα_q/11/12/13_ cell line (red lines) **(C)**, inset: The response in parental and ΔGα_q/11/12/13_ cell lines are shown alone for clarity. Data represent mean ± s.e.m. of [biological replicates performed in triplicate (*n* = 3–15)].

Using a HEK 293 line with deletion of G_q/11_ and G_12/13_ (13), we next assessed how different G proteins might be involved in recruitment of β-arrestin to the FP/AT1R dimer. Ang II-induced recruitment of β-arrestin 1/2-RlucII to the AT_1_R-Venus/FP-WT dimer was measured in the parental and the ΔGα_q/11/12/13_ cell line. The absence of G proteins reduced Ang II-induced response by approximately 50% for β-arrestin 1 and β-arrestin 2 (Figures 5A,B). Individual G proteins were then restored to the ΔGα_q/11/12/13_ cell line to assess which might be able to rescue this loss of β-arrestin recruitment. Replacement of Gα_11_, Gα_12_, or Gα_13_ partially rescued β-arrestin 1 recruitment but surprisingly, not Gα_q_ (Figure 5A). A similar finding was noted for β-arrestin 2 (Figure 5B). Although the data were considerably noisier for the responses measured after treatment with PGF2α, similar patterns were detected for β-arrestin 1 recruitment (Figure 5C, also see *inset*). Interestingly, in this case, re-expression of any of the four Gα subunits was able to partially rescue β-arrestin 2 recruitment mediated by PGF2α (Figure 5D). Although we did not control for expression of the different G protein subunits, these data suggest a complicated interplay between G protein and β-arrestin coupling, inconsistent with a simple interpretation of these events.

### Spatiotemporal Aspects of Differential β-Arrestin 1 Recruitment to the FP/AT1R Heterodimer

**Figure 6:**
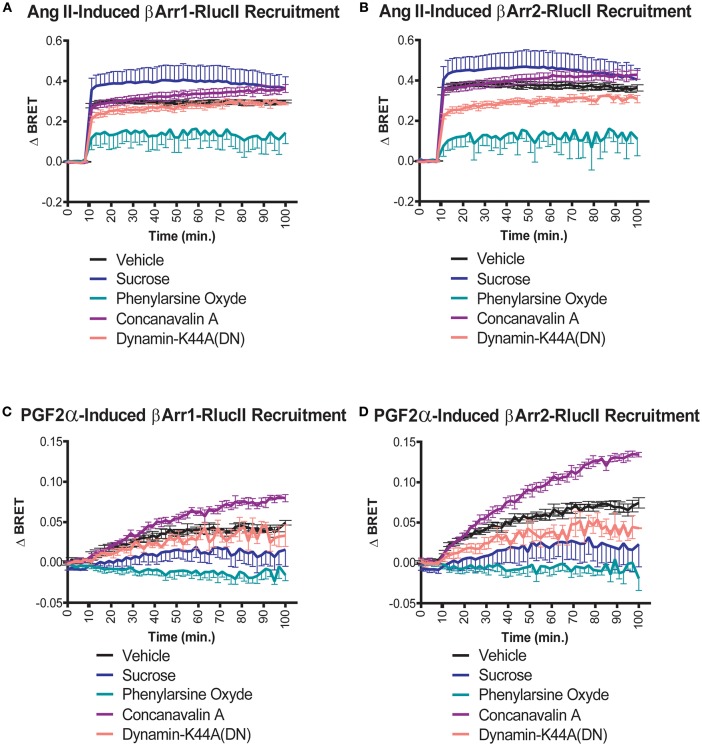
Effects of endocytosis inhibitors on Ang II- and PGF2α-induced β-arrestin 1/2 recruitment to the AT1R/FP dimer. **(A,B)** Sucrose (blue lines, 450 mM) and phenylarsine oxide (blue-green lines, 2.5 μM) pretreatment (30 min) potentiated and diminished the recruitment of β-arrestin1/2-RlucII to the AT1R-Venus/FP-WT dimer in response to Ang II (1 μM), respectively. To a much lesser extent, concanavalin A (purple lines, 0.4 g/L) pretreatment (30 min) and the dominant negative mutant K44A dynamic (salmon lines; 0.5 μg) also potentiated and diminished the responses, respectively. **(C,D)** Concanavalin A (purple lines, 0.4 g/L) and phenylarsine oxide (blue-green lines, 2.5 μM) pretreatment (30 min) potentiated and diminished the recruitments of β-arrestin 1/2-RlucII to the AT1R-Venus/FP-WT dimer in response to PGF_2α_ (1 μM), respectively. To a lower extent, sucrose (blue lines, 450 mM) and the dominant negative mutant K44A of dynamin (salmon lines; 0.5 μg) both diminished the responses. Data represent mean ± s.e.m. of independent experiments performed in triplicate (*n* = 3–4).

We previously showed that stimulation of the receptor pair with either ligand resulted in receptor internalization of the dimer when reconstituted using a split Venus protein complementation (12). The data in that paper, using confocal imaging, hinted that distinct endocytic routes might be taken in response to PGF2α compared to Ang II. Given the role of β-arrestin recruitment in the post-stimulation trafficking of receptors, we examined how different inhibitors of endocytosis affected interactions between the FP/AT1R heterodimer and β-arrestin1/2. As before, we first examined the effects of Ang II on β-arrestin1/2 recruitment. In both cases, neither concanavalin A or a dominant negative dynamin K44 mutant had significant effects compared to control (Figures 6A,B). However, the extent of the recruitment of β-arrestin was decreased significantly by phenylarsine oxide and potentiated by sucrose. The responses to PGF2α were qualitatively different. In this case, again there was no effect of dynamin K44, but both sucrose and phenylarsine oxide reduced β-arrestin1/2 recruitment while concanavalin A potentiated the interaction (Figures 6C,D). These results again suggest that the trafficking itineraries of the heterodimer are distinct following stimulation with either Ang II or PGF2α. Taken together our data suggest that the dimer adopts different fates when stimulated by distinct ligands.

## Discussion

Here, we show that two ligands binding to different sites in a GPCR heterodimer elicit asymmetric responses with respect to β-arrestin1/2 recruitment. Although FP does not normally recruit β-arrestin1/2 (12, 26), as there are GPCRs that don't recruit β-arrestin (29), stimulation of FP can recruit β-arrestin to an AT1R partner in the context of a heterodimer. Further, we show that different ligands acting on receptor dimers may lead to qualitatively distinct β-arrestin1/2 recruitment events with potentially distinct trafficking itineraries. Such observations might help to explain the functional asymmetries we've noted in our previous study (12). Our data add a new wrinkle to signaling via GPCR heterodimers, when we consider how structural and functional asymmetries might play out in terms of bi-directional allosteric receptor regulation.

Previously, we showed that the AT1R and FP formed heterodimers in both HEK 293 and vascular smooth muscle cells (VSMC) where they are endogenously co-expressed. We analyzed several phenotypic responses, including MAPK activation and DNA and protein synthesis in both HEK 293 cells and in VSMC. We showed AT1R when treated with an antagonist strongly potentiated ERK1/2 activation by FP, which was not reciprocated by treatment of FP with its own antagonists when measuring Ang II-mediated ERK1/2 activation (12). We also used [^3^H]-thymidine incorporation as a measure of DNA synthesis, and [^3^H]-leucine incorporation as a measure of protein synthesis and observed that pre-treatment of VSMC with an AT1R antagonist on [^3^H]-thymidine incorporation following PGF2α stimulation could be inhibited by FP blockade, but not by AT1R blockade. However, the FP antagonist was as efficient as the AT1R antagonist in inhibiting both Ang II-induced [^3^H]-thymidine or [^3^H]-leucine incorporation (12). These results highlighted an important asymmetry in the regulation of cellular responses via the receptor heterodimer. This was also reflected in our conformational profiling of both receptors using the FlAsH-BRET approach where conformational information could be transferred from the AT1R through to FP via a shared G protein but the converse could not be detected. Distinct patterns of β-arrestin1/2 recruitment might be at the root of some of the functional asymmetries. Interestingly, a number of GPCR dimers have been demonstrated to recruit β-arrestin1/2 [reviewed in (30)], although whether asymmetries in β-arrestin1/2 recruitment, such as we have measured here have yet to be assessed systematically with respect to other homo- or heterodimers.

Deletion of several Gα subunits significantly abrogated both Ang II- and PGF2α-mediated β-arrestin1/2 recruitment which was in all cases restored by re-expression of Gα_11_, Gα_12_, or Gα_13_ but not always Gα_q_. This is odd, as Gα_q_ is a well-known partner for both receptors. We identified Gα_q/11_ as the conduit by which allosteric information was transferred from AT1R to FP which did not occur in the opposite direction from FP to AT1R (13). Perhaps this is a reflection of a similar asymmetry. It is also one of the rare instances where a distinction could be made between Gα_11_ and Gα_q_. Recent studies have suggested we need to be circumspect about cells gene-deleted for different G proteins that may get “rewired” as a consequence (31). More systematic studies with better controlled levels of G protein expression would be necessary to confirm our findings, but it is clear that changes to β-arrestin1/2 recruitment in the gene-deleted cells used in this study could be partially rescued by restoring individual Gα subunits.

We had previously shown that the trafficking of the putative heterodimer was likely different depending on whether stimulation was via Ang II or PGF2α (12). We noted here that they had distinct sensitivities to different inhibitors of endocytic processes. In response to Ang II, the β-arrestin interaction was inhibited by phenylarsine oxide, a compound whose exact mechanism of action is unknown (32), and potentiated by hypertonic sucrose suggesting that in the latter case, more β-arrestin can be recruited in this case, that is the receptors still remain accessible to β-arrestin because they are not being internalized. Cells treated with phenylarsine oxide might be compromised with respect to G protein dissociation required for β-arrestin recruitment. In response to PGF2α, however, the pattern of inhibition was distinct, suggesting again that receptors were locked into a distinct trafficking itinerary, yielding two distinct fates for the same receptor complex activated by different ligands.

Similar asymmetric structural arrangements were also observed in other GPCR oligomers including the luteinizing hormone receptor (33), rhodopsin (34), mGluR2/3 heterodimers (1), and leukotriene B4 receptor dimers (35). These studies indicate that individual protomers in GPCR dimers and oligomers could interact with shared G proteins via distinct interfaces [see also (6)], providing a mechanism by which such structural asymmetries could lead to asymmetries in receptor conformation and functional outcomes. Such asymmetric conformational crosstalk may provide novel ways to imagine targeting heterodimers, which has been ignored in current drug discovery programs.

Other studies showed that AT1R may be an example of a dimer hub (36) with many different partners such as CB1 cannabinoid receptors (37) apelin receptors, α_2C_AR, and β_2_AR receptor heterodimers (38–42). A more complete exploration of signaling and conformational profiles in GPCR heterodimers would get at the full scope of the impact of receptor protomers on one another.

The AT1R is an important target for treatment of hypertension and heart failure and angiotensin receptor blockers remain widely prescribed (43). A role for FP in the regulation of blood pressure has also been suggested (44). FP has been implicated in parturition with enhanced PGF2α signaling initiating labor through the stimulation of myometrial contraction (45, 46). The AT1R is co-expressed in the myometrium and increased levels have been detected during pregnancy (47–49). Further study of this putative receptor heterodimer could yield novel drug targets for a number of diseases.

## Data Availability

All datasets generated for this study are included in the manuscript and/or the supplementary files.

## Author Contributions

DF and TH designed the study. DF, DD, and RS performed the experiments, analyzed the data, and generated the figures. TH, DF, DD, and AI wrote and edited the paper.

### Conflict of Interest Statement

The authors declare that the research was conducted in the absence of any commercial or financial relationships that could be construed as a potential conflict of interest.

## Abbreviations

GPCR: G protein-coupled receptor
Ang II: angiotensin
AT1R: angiotensin II type I receptor
PGF2α: prostaglandin 2α
FP: prostaglandin 2α receptor
BRET: bioluminescence resonance energy transfer
FlAsH: fluorescein arsenical hairpin binder
RlucII: *Renilla* luciferase II.
